# SARS-CoV-2 infection in cystic fibrosis: A multicentre prospective study with a control group, Italy, February-July 2020

**DOI:** 10.1371/journal.pone.0251527

**Published:** 2021-05-13

**Authors:** Carla Colombo, Gianfranco Alicandro, Valeria Daccó, Vanessa Gagliano, Letizia Corinna Morlacchi, Rosaria Casciaro, Giovanna Pisi, Michela Francalanci, Raffaele Badolato, Elisabetta Bignamini, Barbara Messore, Maria Cristina Lucanto, Giuseppina Leonetti, Massimo Maschio, Marco Cipolli, on behalf of the Italian Cystic Fibrosis Society (SIFC) Study Group for COVID-19

**Affiliations:** 1 Fondazione IRCCS Ca’ Granda Ospedale Maggiore Policlinico, Milan, Italy; 2 Department of Pathophysiology and Transplantation, Università degli Studi di Milano, Milan, Italy; 3 IRCCS Istituto Giannina Gaslini, Genova, Italy; 4 Azienda Ospedaliero-Universitaria di Parma, Parma, Italy; 5 Azienda Ospedaliero-Universitaria Meyer, Firenze, Italy; 6 Clinica Pediatrica e Centro Regionale Fibrosi Cistica Asst Spedali Civili di Brescia, Università degli Studi di Brescia, Brescia, Italy; 7 AOU Città della Salute e della Scienza di Torino SC Pneumologia Presidio OIRM, Torino, Italy; 8 Adult Cystic Fibrosis Centre, Azienda Ospedaliero-Universitaria San Luigi Gonzaga, Orbassano (Torino), Italy; 9 Azienda Ospedaliera Universitaria Policlinico “G. Martino”, Messina, Italy; 10 Azienda Universitaria Ospedaliera Consorziale–Policlinico, Bari, Italy; 11 IRCCS materno infantile Burlo Garofolo, Trieste, Italy; 12 Azienda Ospedaliera-Universitaria Integrata di Verona, Verona, Italy; University of Alabama-Birmingham, UNITED STATES

## Abstract

**Objective:**

To describe the symptoms and clinical course of SARS-CoV-2 infection in patients with cystic fibrosis (CF).

**Methods:**

We carried out a prospective multicentre cohort study based on 32 CF centres and 6597 patients. Centres were contacted to collect baseline and follow-up data of patients who reported symptoms suggestive of COVID-19 or who had contact with a positive/suspected case between the end of February and July 2020. Symptoms and clinical course of the infection were compared between patients who tested positive by molecular testing (cases) and those who tested negative (controls).

**Results:**

Thirty patients were reported from the centres, 16 of them tested positive and 14 tested negative. No differences in symptoms and outcome of the disease were observed between groups. Fever, cough, asthenia and dyspnea were the most frequently reported symptoms. Eight cases (50%) were hospitalized but none required ICU admission. Two adults with a history of lung transplant required non-invasive ventilation, none required ICU admission and all patients fully recovered without short-term sequelae.

**Conclusions:**

The course of SARS-CoV-2 in our patients was relatively favorable. However, COVID-19 should not be considered a mild disease in CF patients, particularly for those with severely impaired respiratory function and organ transplant.

## Introduction

Cystic fibrosis (CF) is a life threatening autosomal recessive disease caused by mutations in the CF transmembrane conductance regulator (CFTR) gene. The encoded protein functions as a chloride channel which regulates the transport of water and electrolytes at the apical membrane of epithelial cells. Lack or dysfunction of the protein leads to abnormalities in ion secretion and absorption and accumulation of thick, sticky secretions in several organs. The major cause of morbidity and mortality is related to lung disease.

In December 2019, a new coronavirus strain named SARS-CoV-2 emerged in Wuhan (China) and the pandemic then spread to Northern Italy [1]. The associated disease was called COVID-19 (Coronavirus Disease 2019) and mainly consisted in severe pneumonia and respiratory illness.

Patients with CF are considered highly vulnerable individuals due to their increased risk of developing severe forms of viral respiratory infections [2].

In this study, we aimed at describing symptoms and clinical course of the infection in a group of CF patients who tested positive for SARS-CoV-2.

## Material and methods

We carried out a prospective multicentre cohort study within the Italian Cystic Fibrosis Society involving 32 CF centres following 6597 patients. Centres were contacted to collect baseline and follow-up data of patients who had reported symptoms suggestive of COVID-19 or who had had contact with a positive/suspected case between the end of February and July 2020.

Patients underwent nasopharyngeal swab followed by molecular testing with polymerase chain reaction (PCR) analysis for SARS-CoV-2 detection and were divided into two groups: those who tested positive (cases) and those who tested negative (controls).

A specifically designed report form was used to collect demographic, clinical, anthropometric, microbiological and respiratory function data, as well as potential epidemiological link of transmission, symptoms, and treatment. The cumulative incidence of SARS-CoV-2 infection was estimated by the ratio between positive cases and the number of patients followed-up in the Italian CF centres. The 95% confidence intervals (95% CI) were computed from the Poisson distribution. The clinical course of the disease was evaluated in terms of vital status, ICU admission, need for oxygen supplementation, non-invasive and invasive ventilation up to October 2020 and also in terms of changes in forced expiratory volume in one second (FEV_1_) from pre-infection to recovery.

Data were compared between groups using Fisher’s exact test for categorical variables and Wilcoxon-Mann-Whitney sum-rank test for continuous variables (two-tailed tests with α = 0.05).

The study was approved by the Ethics Committee of the coordinating centre “Comitato Etico Milano Area 2” and by all the Ethics Committees of the participating centres (Comitato Etico A.O.U. Policlinico G. Martino, Messina; Comitato Etico pediatrico per la Sperimentazione Clinica, Firenze; Comitato Etico Interaziendale A.O.U. San Luigi Gonzaga di Orbassano e AA.SS.LL.TO3– TO4-TO5; Comitato Etico AVEN, Parma; Comitato Etico Regionale della Liguria, Genova; Comitato Etico Interaziendale A.O.U. Città della Salute e della Scienza di Torino—A.O. Ordine Mauriziano—A.S.L. Città di Torino; Ufficio per la qualità della ricerca e la protezione dei soggetti umani dell’IRCCS materno infantile Burlo Garofolo, Trieste; Ethics committee of Brescia; Comitato Etico Indipendente, Azienda Ospedaliero-Universitaria “Consorziale Policlinico” di Bari; Comitato Etico per la Sperimentazione Clinica,CESC, delle Province di Verona e Rovigo). Written informed consent was obtained from each patient or their legal representative.

## Results

Over the study period, the Italian CF centres reported 36 patients with symptoms suggestive of COVID-19 or who had had contact with a positive/suspected case. Among them, 16 patients tested positive by PCR, 15 patients tested negative and 5 patients were not tested. The 5 patients who were not tested and one patient who was negative by PCR but had antibodies against SARS-CoV-2 were excluded since their infection status could not be precisely defined. The final sample consisted of 16 cases (positive by PCR) and 14 controls (negative by PCR). None of the patients in the control group was tested for other viral infections.

**Table 1** provides their baseline characteristics. Controls were older than cases, whereas the two groups were comparable in terms of sex, CFTR genotype, comorbidities, CF-maintenance therapy, and respiratory function prior to the infection. Only one case and one control had severe lung disease before infection, as defined byFEV_1_<40% of predicted. Medical CF management primarily consisted of oral/inhaled antibiotics, azithromycin and oral/inhalation steroids. The most prevalent comorbidities were pancreatic insufficiency, CF-related diabetes and liver disease. Moreover, 11 cases (68.8%) and 10 controls (71.4%) had a chronic/intermittent colonization with *Pseudomonas aeruginosa*. Two cases (12.5%) and one control (7.1%) were lung transplant recipients.

**Table 1:** Baseline characteristics in patients with cystic fibrosis positive (cases) or negative (controls) to molecular test for SARS-CoV-2.

	Cases (N = 16)	Controls (N = 14)	*P* value^a^
**Sex**			1.00
Females	7 (43.8%)	7 (50.0%)	
Males	9 (56.2%)	7 (50.0%)	
**Age (years)**			
Median (range)	20 (0, 57)	24 (2, 52)	0.52
≥ 18 years	8 (50.0%)	12 (85.7%)	0.058
**CFTR genotype**			0.60
F508del heterozygous	6 (37.5%)	7 (50.0%)	
F508del homozygous	3 (18.8%)	1 (7.1%)	
Others	7 (43.8%)	6 (42.9%)	
**z-score of weight-for-length/BMI-for-age,** median (IQR)^b^	0.33 (-0.86, 0.71)	-0.47 (-0.98, 0.34)	0.24
**FEV**_**1,**_ median (IQR)^c^	92 (66, 96)	78 (73, 86)	0.40
**Oxygen therapy**	2 (12.5%)	1 (7.1%)	1.00
***P*. *aeruginosa* infection**	11 (68.8%)	10 (71.4%)	1.00
**Pancreatic insufficiency**	11 (68.8%)	11 (78.6%)	0.69
**CF-related diabetes**	3 (18.8%)	5 (35.7%)	0.42
**CF-related liver disease**	3 (18.8%)	1 (7.1%)	0.60
**Organ transplantation**	2 (12.5%)	1 (7.1%)	1.00
**CF maintenance treatment**			
Antibiotics	10 (62.5%)	8 (57.1%)	0.49
Azithromycin	8 (50.0%)	7 (50.0%)	1.00
Oral/inhalation steroids	9 (56.3%)	10 (71.4%)	0.47
CFTR modulators	2 (12.5%)	2 (14.3%)	1.00
**Contact with a positive/suspected case**	8 (50.0%)	1 (7.1%)	0.017

IQR: interquartile range, FEV_1_: forced expiratory volume in one second.

Data are counts and percentage unless otherwise specified.

^a^ Between-group comparison using Fisher’s exact test for categorical variables and Wilcoxon-Mann-Whitney sum-rank test for continuous variables.

^b^ z-scores of weight-for-length for patients aged ≤ 2 years (2 cases and 1 control) and BMI-for-age for patients aged > 2 years were computed using WHO reference data.

^c^ 3 patients (2 cases and 1 control) were too young to perform spirometry.

Eight cases (50%) had a clearly documented contact with a SARS-CoV-2 positive person and three of them were asymptomatic. The cumulative incidence of SARS-CoV-2 infection was 2.4/1,000 patients (95% CI: 1.4–3.9).

**Table 2** shows the distribution of COVID-19 suggestive symptoms, treatment and clinical course of infection in cases and controls. Fever, cough, asthenia and dyspnea were the most frequently reported symptoms. The median duration of symptoms was 14 days in the case group and 8 days in the control group.

**Table 2:** Symptoms, treatment and outcome in patients with cystic fibrosis positive (cases) or negative (controls) to molecular test for SARS-CoV-2.

	Cases (N = 16)	Controls (N = 14)	*P* value^a^
**COVID-19 symptoms**			
Fever	10 (62.5%)	12 (85.7%)	0.23
Cough	10 (62.5%)	9 (64.3%)	1.00
Dyspnea	7 (43.8%)	5 (35.7%)	0.72
Increased sputum production	1 (6.2%)	0	1.00
Asthenia	7 (43.8%)	7 (50.0%)	1.00
Headache	3 (18.8%)	1 (7.1%)	0.60
Joint pain	1 (6.2%)	4 (28.6%)	0.16
Pharyngodynia	0 (0%)	4 (28.6%)	0.04
Anosmia/Dysgeusia	0	1 (7.1%)	0.60
Diarrhea	2 (12.5%)	0	0.49
Vomit	1 (6.3%)	0	1.00
Hemoptysis	1 (6.3%)	0	1.00
Asymptomatic	3 (18.8%)	0	0.23
**Duration of symptoms** (days)^b^			
Median (range)	14 (2, 54)	8 (1, 54)	0.33
**COVID-19 treatment**			
Lopinavir/ritonavir	3 (18.8%)	0	0.23
Darunavir /Cobicistat	1 (6.3%)	0	1.00
Hydroxy-chloroquine	3 (18.8%)	0	0.60
Azithromycin	3 (18.8%)	0	0.23
Other antibiotics	11 (68.8%)	12 (85.7%)	0.40
**Course of infection**			
Hospitalization	8 (50.0%)	8 (57.1%)	0.73
Admission in ICU	0	0	-
Additional or continuous need of oxygen therapy^c^	3 (18.8%)	4 (28.6%)	0.67
Non-invasive ventilation	2 (12.5%)	0 (0%)	0.49
Invasive ventilation	0	0	-
Change in FEV_1,_ median (IQR)^d^	3.0 (-1.5, 5.5)	-3.0 (-8.5,6.3)	0.48
Clinically recovered	16 (100%)	14 (100%)	-
Died	0	0	-

IQR: interquartile range, FEV_1_: forced expiratory volume in one second

Data are counts and percentage unless otherwise specified.

^a^ Between-group comparison using Fisher’s exact test for categorical variables and Wilcoxon-Mann-Whitney sum-rank test for continuous variables.

^b^ 3 cases were asymptomatic.

^c^ 1 case and 3 controls required additional oxygen therapy during the infection.

^d^ Changes from pre-infection (average value over the 3 months before the PCR test) to recovery (∼2 months after the end of symptoms). Data not available in 6 patients (2 cases and 4 controls).

Eight cases (50%) were hospitalized but none required ICU admission. Two adult males with a history of lung transplant, under immunosuppressive therapy, and with several comorbidities required additional non-invasive ventilation.

**Fig 1:**
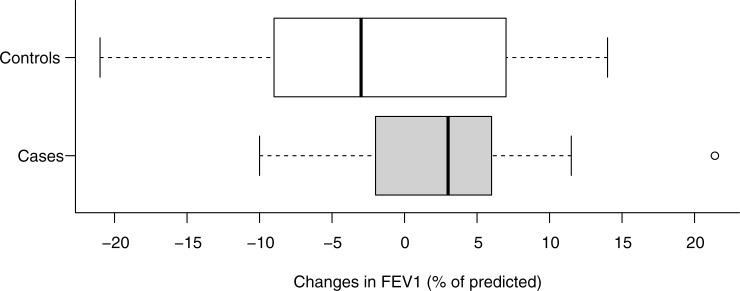
Changes in FEV_1_ (% of predicted) among patients who tested positive for SARS-CoV-2 (cases) by PCR analysis and those who tested negative (controls)^a^. ^a^ Changes from pre-infection (average value over the 3 months before the PCR test) to recovery (∼2 months after the end of symptoms). Data not available in 6 patients (2 cases and 4 controls).

Changes in FEV_1_ did not significantly differ between groups (**Fig 1**). One case and two controls had a reduction in FEV_1_ of 10% and over.

No deaths were observed in either group, and all patients fully recovered without short-term sequelae.

## Discussion

Overall, CF patients with Sars-CoV-2 infection included in our study had a relatively favorable course.

Our results are in agreement with a multinational report across 8 countries which enrolled 40 CF patients positive for SARS-CoV-2 during the early time of the pandemic (between February and mid-April 2020) [3]. In that study, 13 patients required oxygen therapy, one patient required invasive ventilation, and despite 4 patients were admitted to ICU, no deaths were reported and, by the time the study ended, 70% of them fully recovered.

Symptoms were quite similar to those reported by the control group, were mostly non-specific, and could have been easily mistaken for a typical pulmonary exacerbation.

Most CF cases contracted the infection from household members, with a lower incidence compared to the general population (2.4/1,000 CF patients vs. a 4.1/1,000 inhabitants registered among the Italian population by the end of July [4]). A lower incidence was also observed in two other European national studies: one from Spain which reported only 8 cases of SARS-CoV-2 infection between March and mid-May 2020, with a cumulative incidence of 3.2/1,000 among CF patients and 4.9/1,000 in the Spanish population [5]; the other from France [6], the latter suggesting that between March and June 2020 the risk of infection in CF patients was 93% lower than that expected if CF patients had the same risk of the general French population of the same age [6]. It is likely that the habitual use of face masks, hand hygiene, and reduced physical contacts, together with early adoption of “shielding” behaviors during the pandemic, have contributed to contain the infection. Additionally, CF patients receive chronic treatment with azithromycin because of its anti-inflammatory properties in airway epithelial cells, however, the antiviral and immunomodulating properties of this drug remain uncertain [7, 8].

Our study provides evidence that the clinical course of SARS-CoV-2 infection did not substantially differ from other respiratory tract infections. The inclusion of a control group allowed for the first time a direct evaluation of the impact of SARS-CoV-2 infection on CF patients, and is certainly the main strength of our study.

Only a few of our cases were treated with antivirals and hydroxyl-chloroquine, which were the drugs recommended at the time of infection. The clinical course was milder than expected, as a likely consequence of some potential protective factors for severe presentation, such as the young age of the patients, the rarity of cardiovascular risk factors [9], as well as the CF-characteristic respiratory microbiota [10], which may have promoted a more effective immune response to viral infection [11].

The baseline lung inflammatory status may have modulated the individual susceptibility to SARS-CoV-2. Particularly interesting in this regard is the observation of a chronic presence of neutrophils in CF lung that may indeed play an essential role in the control of infections and in tissue repair [12]. Neutrophils are able to counteract infections through phagocytosis and/or the release of neutrophil extracellular traps that may contribute to modulate the individual susceptibility to SARS-CoV-2 [13]. In the present study, however, sputum inflammatory markers were not assessed.

The protective role of chronic inflammation and neutrophil presence may also at least in part, explain the fact that in our study SARS-CoV-2 positive cases were younger than controls. However, the higher frequency of contacts among adolescents and the more prudent behaviors among older patients may also have contributed to the observed age difference.

P. *aeruginosa* infection in CF patients is a common finding and about 70% of cases and controls in our study had positive sputum cultures for *P*. *aeruginosa* and were treated aggressively with IV antibiotics, as per standard practice. Bacterial superinfection, with *P*. *aeruginosa* has been associated with longer duration of ventilation in critically ill COVID-19 patients [14], but this was not the case for our patients, none of whom required mechanical ventilation.

Our study has some limitations. First, during the study period only a small number of patients contacted the CF centers with symptoms suggestive of COVID-19, as a likely consequence of a general reduction of respiratory infections induced by the restrictive measures adopted. In addition, many symptomatic patients were treated with antibiotic therapy at home and thus did not access the CF centre. This may have eventually introduced a selection of controls with more severe symptoms who could not be treated at home. However, the favorable clinical course in controls does not support a selection bias.

We cannot rule out that the testing strategy during the first phase of the pandemic, with only symptomatic patients being tested by PCR and limited use of serological tests, could have led to an underestimation of the real infection rate. However, seroprevalence data from Belgium seem to confirm the low infection rate in the CF population [15].

The role of other viral respiratory infections could not be ascertained. Future studies should explore this important issue to untangle the differential responses to SARS-CoV-2 infection vs other common viral infections that trigger hospitalization in CF patients.

Finally, our data refer only to the first phase of the pandemic, where SARS-CoV-2 mostly hit the Northern regions of the country, while from September 2020 it spread rapidly all over Italy. At that time only the original SARS-CoV-2 strain was circulating, and it cannot be excluded that the new current circulating variants may have different impacts on this patient population [16].

Despite the apparently lower infection rate, CF patients should continue to follow public health advices and protect themselves from SARS-CoV-2 infection [17] since data from an international network based on 181 confirmed cases reported up to mid-June 2020 from 38 countries, indicates that COVID-19 cannot be considered a mild disease [18]. As for 29 December 2020, 234 cases were registered, 13 of them requiring intensive care and 5 who died [19].

Further research is therefore needed to better understand the long-term implications of this new pathogen in the CF population, especially in those at higher risk because of severe respiratory impairment or history of organ transplant.

## Supporting information

S1 Data (XLSX)

## Abbreviations

CF: Cystic Fibrosis
CFTR: Cystic Fibrosis Transmembrane Conductance Regulator
CI: Confidence Interval
COVID-19: Coronavirus Disease 2019
FEV1: Forced Respiratory Volume in one second
PCR: Polymerase Chain Reaction
SARS-CoV2: Severe Acute Respiratory Syndrome Coronavirus 2
